# Variation in *Peperomia pellucida* growth and secondary metabolism after rhizobacteria inoculation

**DOI:** 10.1371/journal.pone.0262794

**Published:** 2022-01-21

**Authors:** Nayara Sabrina Freitas Alves, Suzana G. Kaory Inoue, Adriana Ribeiro Carneiro, Ulisses Brigatto Albino, William N. Setzer, José Guilherme Maia, Eloisa Helena Andrade, Joyce Kelly R. da Silva

**Affiliations:** 1 Programa de Pós-Graduação em Biotecnologia, Universidade Federal do Pará, Belém, Brazil; 2 Faculdade de Biotecnologia, Universidade Federal do Pará, Belém, Brazil; 3 Instituto de Ciências Exatas, Universidade Federal do Sul e Sudeste do Pará, Marabá, Brasil; 4 Department of Chemistry, University of Alabama in Huntsville, Huntsville, AL, United States of America; 5 Aromatic Plant Research Center, Lehi, UT, United States of America; 6 Programa de Pós-Graduação em Química, Universidade Federal do Pará, Belém, Brazil; Tocklai Tea Research Institute, INDIA

## Abstract

*Peperomia pellucida* L. Kunth is a herb well-known for its secondary metabolites (SM) with biological potential. In this study, the variations in the SM of *P*. *pellucida* during association with rhizobacteria were evaluated. Plants were inoculated with *Enterobacter asburiae* and *Klebsiella variicola*, which were identified by sequencing of the 16S rRNA gene. The data were evaluated at 7, 21, and 30-day post inoculation (dpi). Plant-bacteria symbiosis improved plant growth and weight. Total phenolic content and phenylalanine ammonia lyase enzyme activity had a significant increase mainly at 30 dpi. *P*. *pellucida* was mainly composed of phenylpropanoids (37.30–52.28%) and sesquiterpene hydrocarbons (39.28–49.42%). The phenylpropanoid derivative 2,4,5-trimethoxy-styrene (ArC2), the sesquiterpene hydrocarbon ishwarane, and the phenylpropanoid dillapiole were the major compounds. Principal component analysis (PCA) of the classes and compounds ≥ 2.0% indicated that plants colonized by *E*. *asburiae* had a reduction in the content of sesquiterpene hydrocarbons and an increase in phenylpropanoids and derivatives. Plants treated with this bacterium also had an increase in the content of 2,4,5-trimethoxystyrene at 30 dpi. Plants inoculated with *K*. *variicola* had significant increases only in the content of the classes monoterpene hydrocarbons and ‘other compounds’ (hydrocarbons, esters, ketones, etc.). These data suggest that the production of plant secondary metabolites can be modified depending on the type of rhizobacteria inoculated.

## 1. Introduction

Piperaceae is a family of angiosperms composed of approximately 3,700 species from which 2,000 species belong to the genus *Piper* and 1,600 to the group *Peperomia*. The species *Peperomia pellucida* (L.) Kunth can be found in the Neotropics, Africa, Southeast Asia, and Oceania [1]. It is well-known for its biological activities such as cytotoxicity (leukemia HL-60, cervical HeLa, and breast MCF-7) [2], fracture healing [3], analgesic [4], antimicrobial [5] and anti-inflammatory [6]. In addition, the plant is used in popular medicine to treat inflammation, hypertension, cough, cardiac arrhythmia, skin bruises, and several other health problems [1].

*Peperomia pellucida* essential oil (EO) is mainly characterized by the presence of dillapiole (20.7–55.3%) [7,8]. Plants from the Brazilian Amazon, for example, are mostly composed of dillapiole (39.7–55.3%), β-caryophyllene (10.7–14.3%) and carotol (0–8.1%) [7,9]. Its extracts are dominated by phenylpropanoid pathway derivatives with a wide variety of lignans with different skeletons [1,10]. The biosynthesis of secondary metabolites can be influenced by physiological and environmental factors such as temperature, seasonality [11], type of soil [12], salinity [13], and symbiosis with microorganisms [14,15].

Plant growth-promoting rhizobacteria (PGPR) flourish in the rhizosphere of plants by growing inside or around their tissues. These microorganisms are well-known for their potential to fix atmospheric nitrogen [16], solubilize phosphorus and produce siderophores that sequester iron [17], including species of the genera *Pseudomonas*, *Bacillus*, *Enterobacter* and *Klebsiella* (Section 4). PGPR can act as biofertilizers, increasing the availability and absorption of important minerals [18], and in the biological control of phytopathogens [19] and production of phytohormones [20].

In addition, plant association with bacteria can affect plant secondary metabolism. Specifically, these microorganisms can alter plant essential oil yield and composition, as well as its non-volatile compounds [21,22]. Seedlings of *Origanum majorana* L. inoculated with *Pseudomonas fluorescens* had an increase in essential oil (EO) yield from 0.05% to 0.14% [23]. Leaf spraying and micro-injection of *P*. *fluorescens* and *P*. *aeruginosa* in chickpeas (*Cicer arietinum*) infected with *Sclerotinia sclerotiorum* induced phenylalanine ammonia-lyase (PAL) activity and phenolic compounds [24].

Therefore, this study aimed to evaluate changes in the secondary metabolism of *P*. *pellucida* during association with rhizobacteria. The bacterial strains EM56 and EM09 were isolated from the rhizosphere of *Schizolobium amazonicum* Huber ex Ducke (State of Pará, Amazon region, Brazil) and identified by sequencing of the 16S rRNA gene as *Enterobacter asburiae* strain EM56 and *Klebsiella variicola* strain EM09. These microorganisms were used in this study because of their potential for nitrogen fixation, phosphate solubilization and plant-growth promotion [25–28]. Additionally, plant inoculation with PGPR can improve both plant growth and change plant secondary metabolism depending on the bacterial species inoculated.

## 2. Materials and methods

### 2.1 Plant sample collection and experimental procedure

*Peperomia pellucida* plants (30–40 days old) were collected at the Universidade Federal do Pará (UFPA), campus Belém, Pará, Brazil, in the location site 1 (UTM coordinate: 22S 9837030 782873) and 2 (UTM coordinate: 22S 9836945 783313). A collection authorization was not required since it is an invasive plant which was not collected in a protected area. The experiment was conducted in a greenhouse located in the Institute of Biological Sciences (ICB) of UFPA under 70% of shading, in the same coordinate of location 1. Inoculated (PpI) and control (PpC) plants were named according to the day of collection: PpI-7 and PpC-7; PpI-21 and PpC-21; and PpI-30 and PpC-30. All analyses were performed at 7, 21 and 30 days post inoculation (dpi). The experiment was conducted according to the following Sections.

### 2.2 Bacteria isolation

The bacterial strains EM09 and EM56 were isolated from the rhizosphere of ‘paricá da Amazônia’ (*Schizolobium amazonicum* Huber ex Ducke) present in forest fragments of the territory Transamazônica-Xingu, State of Pará, Brazil. Paricá plants (30–40 cm long) were collected with roots and root soil, stored in plastic bags and maintained on ice. In the laboratory, 10 g of soil was diluted in 9 mL of sterile 0.85% NaCl solution (Sigma-Aldrich Brasil Ltda, São Paulo, Brazil), and 1 mL was used in a serial dilution of 10^−4^. Around 50 μL from the forth dilution was plated in two Petri dishes containing Luria-Bertani (LB) agar medium (Sigma-Aldrich Inc., St. Louis, Missouri, USA), using the spread plating method in a vertical laminar flow hood under sterile conditions [29,30].

Plant roots were washed with tap water to remove soil, cut into small pieces of approximately 2 cm, and immersed in 70% ethyl alcohol for 5 minutes to eliminate the external microbiota. The material was rinsed 6 times with sterile distilled water, and 10 g macerated with a sterile glass rod in test tubes containing 9 mL of sterile 0.85% NaCl solution. The material was submitted to a serial dilution of 10^−4^ and plated as previously described. Plates were incubated at 28°C for 24 h [29,30]. All bacteria isolation experiments were performed in biological triplicates.

### 2.3 DNA extraction, 16S gene PCR and sequencing

The bacterial strains *E*. *asburiae* and *K*. *variicola* were grown in 10 mL of LB liquid medium and incubated at 28°C for 24 h. The microbial suspensions were centrifuged at a gravitational force (Force G) of 6000 g at 4°C for 10 min to obtain the pellet that was used for DNA extraction by the DNeasy Blood and Tissue Kit (Qiagen^®^). The 16S rRNA gene was amplified using the universal primers 8F (5’AGAGTTTGATCCTGGCTCAG 3’) and 1492R (5’ TACGGYTACCTTGTTACGACTT 3’). PCR was carried out in 50-μL reaction mixtures containing 0.2 mM of DNTP solution, 1.5 mM of MgCl_2_ solution, 0.2 pmol of each primer, 1 U of Taq DNA polymerase (Invitrogen, Carlsbad, California, USA) and 50 ng of DNA. Cycling conditions had an initial denaturation step (95°C, 5 min) followed by 35 cycles of annealing (95°C, 1 min), extension (60°C, 1 min) and denaturation (72°C, 1 min). The process was terminated by a final extension step (72°C, 7 min) [31].

Sequencing was performed by the Sanger method at ACTGene Análises Moleculares, Rio Grande do Sul, Brazil, with a 50 cm capillary sequencer and AB 3500 platform. The sequences were visualized in the program BioEdit and identified through local alignment using the National Center for Biotechnology Information (NCBI) tool named BLAST. The evolutionary history of the strains was evaluated using the program MEGA6 [32] by the neighbor-joining method [33]. The phylogeny test applied was the Bootstrap and the evolutionary distances were calculated by the *p*-distance model [34].

### 2.4 Preparation of bacterial inoculum

The strains *E*. *asburiae* and *K*. *variicola* were individually cultivated in 300 mL of LB medium for 4 h at 28°C. Approximately 1 mL of each microbial culture was regularly collected from the Erlenmeyer flask in a laminar flow hood and the density measured until the absorbance at 600 nm (OD_600_) reached 0.4, which contained around 10^8^ Colony Forming Units (CFU) per mL [35]. The grown medium was centrifuged at 7600 g for 30 min, the supernatant discarded and the pellet homogenized with 300 mL of sterile 0.85% NaCl solution containing 0.5% of cellulose [36].

### 2.5 Plant cultivation and inoculation

*Peperomia pellucida* cuttings containing 3 or 4 nodes and ½ of a leaf were propagated in sterile vermiculite type B (Urimamã Mineração Ltda, Santa Maria da Boa Vista, Brazil). Nutrient solution (Biofert Root) containing N, P_2_O_5_, K_2_O, S, B, Cl, Cu, Fe, Mn, Mo and Zn was applied at every 15 days. After 30 days of cultivation, plants were inoculated with 5 mL of the bacterial inoculum 3 to 5 cm below the soil surface. Control plants (non-inoculated with bacteria) received only 5 mL of sterile 0.85% NaCl solution with 0.5% cellulose (Section 2.3) [37].

The experiment was performed in vermiculite since plants were supplemented with nutrient solution containing nutrients also important to the microorganisms studied. This strategy has also been used in other similar studies with herbs since their growth conditions are harder to be reproduced in a greenhouse [21,38–40].

### 2.6 Plant development evaluation

The following plant growth parameters were evaluated: number of leaves, number of nodes, height (cm), root length (cm), leaves and roots fresh biomass (g). Height was measured from the plant collar to the end of the terminal bud of the main branch. Leaf and node numbers were counted in the collection site. Plant leaves and roots were collected in aluminum foil, maintained on ice, weighted and conserved under refrigeration at -20°C.

### 2.7 Extraction and analysis of volatile compounds

Leaf volatile compounds were extracted by simultaneous distillation using the Likens-Nickerson extractor for 2 h with 3 mL of *n*-pentane. Aliquots of 1 μL of the resulting organic fraction were analyzed by GC-MS. The qualitative analysis was carried out on a Shimadzu QP2010 plus instrument under the following conditions: Rtx 5MS silica capillary column (30 m × 0.25 mm × 0.25 μm); programmed temperature of 60–240°C (3°C/min); carrier gas helium with velocity of 32cm/s; type of injection splitless and ionization by electronic impact (70 eV); injector temperature of 250°C; ion source and transfer line temperature of 200°C. The identification of compounds was performed by comparison of mass spectrum and retention index (RI) with data present in the libraries NIST [41] and Adams [42]. The RIs were calculated using a homologous series of *n*-alkanes (C8–C20, Sigma–Aldrich) [43].

### 2.8 Determination of total phenolic content (TPC)

The extract fractions from fresh leaves (2 g) were obtained by percolation (96 h) with 50 mL of methanol. After solvent evaporation, the Folin-Ciocalteu method was used to determine total phenolic content (TPC) [44]. The extracts were solubilized again in methanol at a concentration of 20 mg/mL and then diluted 30 times in water because of our samples reactivity. This dilution should be tested for each type of plant sample in order to maintain an absorbance between 0.3 and 0.7. Aliquots of 500 μL of the diluted sample received 250 μL of Folin-Ciocalteu (1 N) and then 1,250 μL of Na_2_CO_3_ (75 g/L). After 30 min of incubation in the dark, the absorbance was read at 760 nm using a UV-Vis spectrophotometer (Ultrospec 5300 pro, Amersham Biosciences, Little Chalfont, Reino Unido). The experimental calibration curve was prepared using gallic acid. TPC was expressed as milligrams of gallic acid equivalents (GAE) per gram of extract (mg/GAE g^−1^) [44].

### 2.9 In vitro phenylalanine ammonia-lyase (PAL) activity

Leaves were frozen and then macerated in liquid nitrogen. An amount of 250 mg of macerated leaves was homogenized in 1 mL of sodium borate buffer solution (0.3 mM, pH 8.8), 1 mM EDTA, 1 mM DTT and 5% polyvinylpolypyrrolidone. The material was centrifuged at 13,000 g for 20 minutes at 4°C. An aliquot of 0.5 mL of the supernatant was mixed with 1 mL of 0.3 mM sodium borate buffer at pH 8.8 with 0.03 mM L-phenylalanine and incubated for 15 minutes at 25°C. The activity was evaluated in a UV-Visible spectrophotometer at 290 nm by quantification of (*E*)-cinnamic acid produced from L-phenylalanine. The blank had only the sodium borate buffer with L-phenylalanine and water [45]. The molar extinction coefficient of (*E*)-cinnamic acid (9630 mol·L^−1^·cm^−1^) was applied to determine the enzyme activity [45,46].

### 2.10 Statistical analysis

The experiment was performed in completely randomized blocks with 20 plants for treatment with a total of 120 individuals (60 controls and 60 inoculated). All analyses were performed in triplicate, compared with the control group and expressed as means ± standard deviation. Analyses of variance of plant developmental parameters, PAL enzyme activity, TPC and major volatile compounds were conducted by Bonferroni test, Two-way ANOVA, using the software GraphPad Prism 7.0. Differences at *p* <0.05 were considered statistically significant.

Volatile compounds were also submitted to a multivariate analysis using as variables the components with percentages ≥ 2.0% and the total sum of the classes of compounds (monoterpene hydrocarbons, sesquiterpene hydrocarbons, oxygenated sesquiterpenes, phenylpropanoids and derivatives, and other compounds). The data matrix was standardized by subtracting the mean from each value and dividing it by the standard deviation. Principal Component Analysis (PCA) was performed in the Software Minitab (free version 390, Minitab Inc., State College, PA, USA) [47–49].

## 3. Results

### 3.1 Identification of the bacteria by 16S rRNA gene sequencing

**Fig 1:**
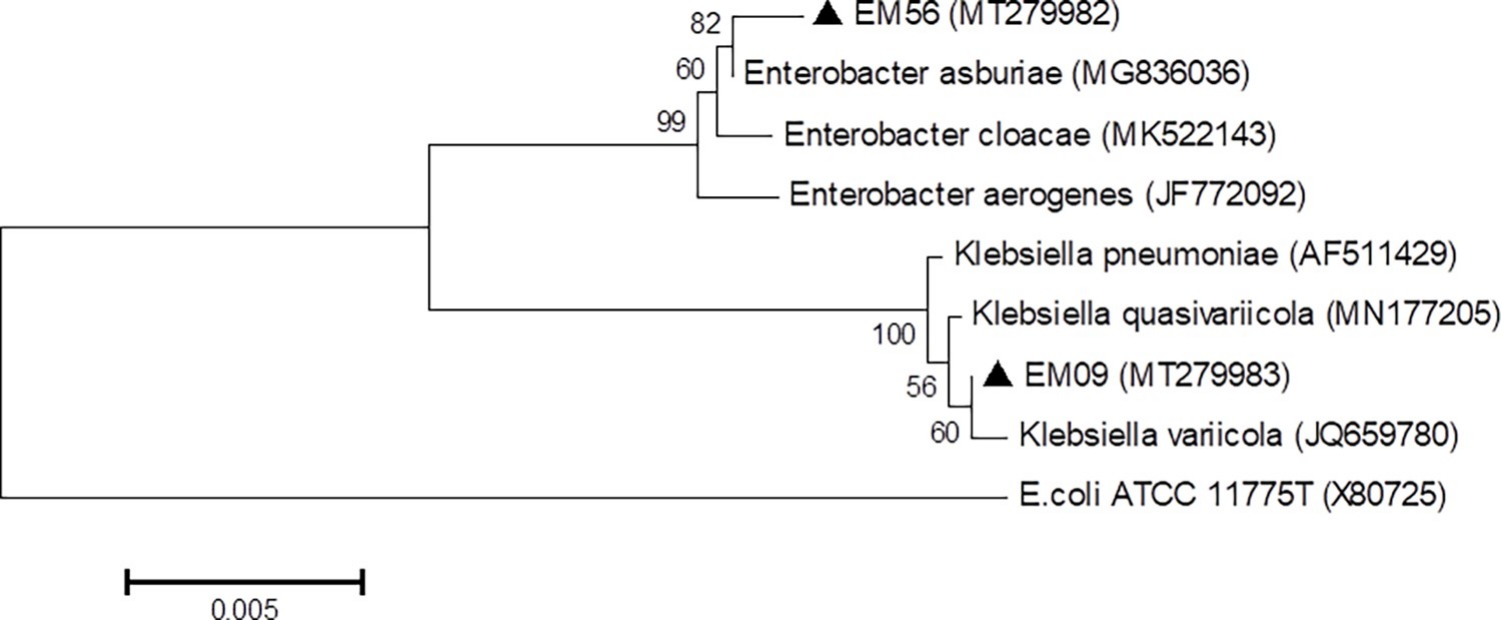
Phylogenetic tree of the bacteria *Enterobacter asburiae* strain EM09 and *Klebsiella variicola* strain EM56 based on sequencing of the 16S rRNA gene. The analysis contained 9 nucleotide sequences with 1,354 positions in the final data set. The *Escherichia coli* sequence was used as an external group. The scale bar represents approximately 5 base substitutions per 1000 nucleotide positions.

The bacteria EM56 and EM09 were isolated from roots and soil of *paricá*. The 16S rRNA gene was amplified by PCR and the amplicon sequenced. EM56 and EM09 had a sequence size of 1359 pb and 1393 pb, respectively, which were deposited in the GenBank and assigned with the accession numbers MT279982 and MT279983. Both DNA sequences were submitted to a search for homology on the tool BLAST of the NCBI. EM56 showed 99.85% of similarity with *Enterobacter asburiae* and isolate EM09 indicated 99.93% of similarity with *Klebsiella variicola*. The phylogenetic analysis was performed to show the bacterial species and their relation with other microorganisms (Fig 1). Both microorganisms are well-known for their potential for nitrogen fixation, phosphate solubilization and siderophore production [27,50].

### 3.2 Comparative analysis of plant development

Plants inoculated with *E*. *asburiae* (Table 1) displayed an increase of 24.82% and 34.48% in the number of leaves and leaf weight at 21 dpi, respectively. Nonetheless, at 30 dpi there was a reduction of 13.20% in the number of leaves and an increase of 61.10% in their weight since they became larger. There was a rise in the number of nodes at 21 and 30 dpi (46.70% and 32.40%). However, no significant change was found for plant height, which indicates that inoculated plants had shorter internodes. The parameter root length was not affected by bacteria colonization, but root weight increased by 66.70% at 21 dpi and 85.70% at 30 dpi.

**Table 1:** Developmental parameters of *Peperomia pellucida* after inoculation with *Enterobacter asburiae*.

dpi	Treatment	Evaluation parameters ^‡^											
		Leaves (n^o^)	CV value (%)	Nodes (n^o^)	CV value (%)	Height (cm)	CV value (%)	Root (cm)	CV value (%)	Fresh weight–leaves (g)	CV value (%)	Fresh weight–root (g)	CV value (%)
**07**	**PpC**	24.3±1.8	7.3	17.0±1.2	7.2	20.1± 1.2	5.8	9.8±0.8	8.3	0.6± 0.1	8.6	0.2±0.1	35.5
	**PpIE**	26.3±4.5	17.1	19.7±1.2	6.3	18.3±1.0	5.6	11.5±0.8	6.5	1.4± 0.3*	23.3	0.3±0.1	19.5
**21**	**PpC**	71.7±4.2	5.8	46.0± 2.2	4.7	36.8±0.8	2.3	13.3±1.3	9.8	2.9 ±0.1	2.7	0.9± 0.1	6.1
	**PpIE**	89.5±0.4*	0.5	67.5±1.2*	1.8	34.5±3.4	9.8	14.8±1.0	6.9	3.9±0.1*	2.7	1.5±0.4*	29.4
**30**	**PpC**	75.7±3.3*	4.4	39.5±2.9	7.2	36.8±2.5	6.7	14.8±1.4	9.7	1.8±0.3	17.4	0.7±0.1	20.1
	**PpIE**	65.7± 2.4	3.6	52.3±2.4*	4.5	36.7±0.6	1.7	15.2±2.0	13.3	2.9±0.2*	7.6	1.3±0.2*	13.3
**DF values**		2.0	2.0	2.0	2.0	2.0	2.0	2.0	2.0	2.0	2.0	2.0	2.0

^**‡**^mean ± standard deviation (n = 3).

*Statistically different according to Bonferroni-test (*p* < 0.05). **dpi**: Days post inoculation. **PpC**: *P*. *pellucida* control. **PpIE**: *P*. *pellucida* inoculated with *Enterobacter asburiae*. CV: Coefficient of variation. DF: Degree of freedom. Note: Each variable is followed by its CV value.

Plants inoculated with *K*. *variicola* exhibited an increase of 32.80% and 19.80% in the number of leaves at 21 and 30 dpi, respectively (Table 2). However, leaf weight was only improved at 30 dpi (50.0%). Furthermore, the number of nodes increased by 52.80% at 21 dpi and by 75.60% at 30 dpi. Height was improved in all days analyzed (18.30%, 30.30% and 25.0%). There was also a 33.30% increase in root length and a 200.0% increase in root weight at 21 dpi. No significant changes were observed at 30 dpi.

**Table 2:** Developmental parameters of *Peperomia pellucida* after inoculation with *Klebsiella variicola*.

dpi	Treatment	Evaluation parameters ^‡^											
		Leaves (n^o^)	CV value (%)	Nodes (n^o^)	CV value (%)	Height (cm)	CV value (%)	Root (cm)	CV value (%)	Fresh weight–leaves (g)	CV value (%)	Fresh weight–root (g)	CV value (%)
**07**	**PpC**	24.0±1.6	6.8	17.0±1.4	8.3	17.5±1.1	6.2	8.3±0.2	2.8	1.1±0.1	9.5	0.2± 0.03	14.2
	**PpIK**	31.7±0.5	1.5	22.7±1.2	5.5	20.7±1.3*	6.4	10.4±0.1	0.9	1.9±0.2	8.8	0.4±0.1	19.0
**21**	**PpC**	53.7±2.9	5.3	43.0±7.9	18.3	19.8±0.2	1.0	10.2±1.9	19.0	2.0±0.1	4.4	0.2±0.2	10.4
	**PpIK**	71.3±5.4*	7.6	65.7±8.5*	12.9	25.8 ±0.5*	1.8	13.6±1.6*	11.8	2.8±0.5	19.0	0.6±0.1*	15.5
**30**	**PpC**	76.3±10.6	13.9	39.7±7.3	18.4	26.8±1.3	5.0	11.2±0.5	4.3	2.8±0.1	2.3	0.6±0.1	18.9
	**PpIK**	91.4±2.7*	3.0	69.7±4.0*	5.8	33.5±0.4*	1.2	13.3±0.5	3.5	4.2±0.8*	17.8	0.8±0.2	23.8
**DF values**		2.0	2.0	2.0	2.0	2.0	2.0	2.0	2.0	2.0	2.0	2.0	2.0

^**‡**^mean ± standard deviation (n = 3).

*Statistically different according to Bonferroni-test (*p* < 0.05). **dpi**: Days post inoculation. **PpC**: *P*. *pellucida* control. **PpIK**: *P*. *pellucida* inoculated with *Klebsiella variicola*. CV: Coefficient of variation. DF: Degree of freedom. Note: Each variable is followed by its CV value.

### 3.3 Phenylalanine ammonia-lyase (PAL) activity

**Fig 2:**
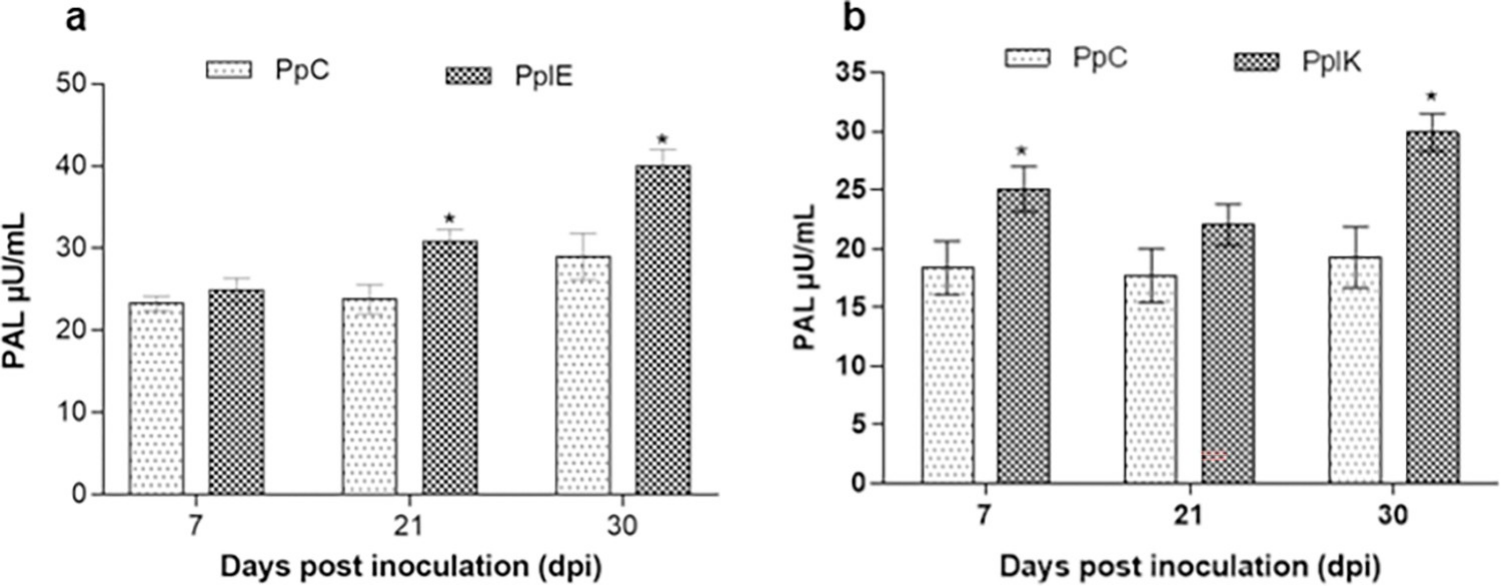
Variation in the PAL enzyme activity in plants inoculated (n = 3) with *Enterobacter asburiae* (A) and *Klebsiella variicola* (B). *Statistically different according to Bonferroni test (*p* < 0.05). **dpi**: Days post inoculation. **PpC**: *P*. *pellucida* control. **PpIE**: *P*. *pellucida* inoculated with *Enterobacter asburiae*. **PpIK**: *P*. *pellucida* inoculated with *Klebsiella variicola*.

Plant inoculation induced a higher production of PAL enzyme in the leaves. Propagules colonized by *E*. *asburiae* had an increase in the unit of enzyme/mL of extract of 34.0% and 38.0% at 21 and 30 dpi (23.0–30.80 and 29.0–40.0 μU/mL, respectively) (Fig 2A). Similarly, herbs treated with *K*. *variicola* had a rise of 36.80% (18.35–25.10 μU/mL) and 55.32% (19.25–29.90 μU/mL) in the enzyme activity at 7 and 30 dpi, respectively (Fig 2B).

### 3.4 Total phenolic determination

**Fig 3:**
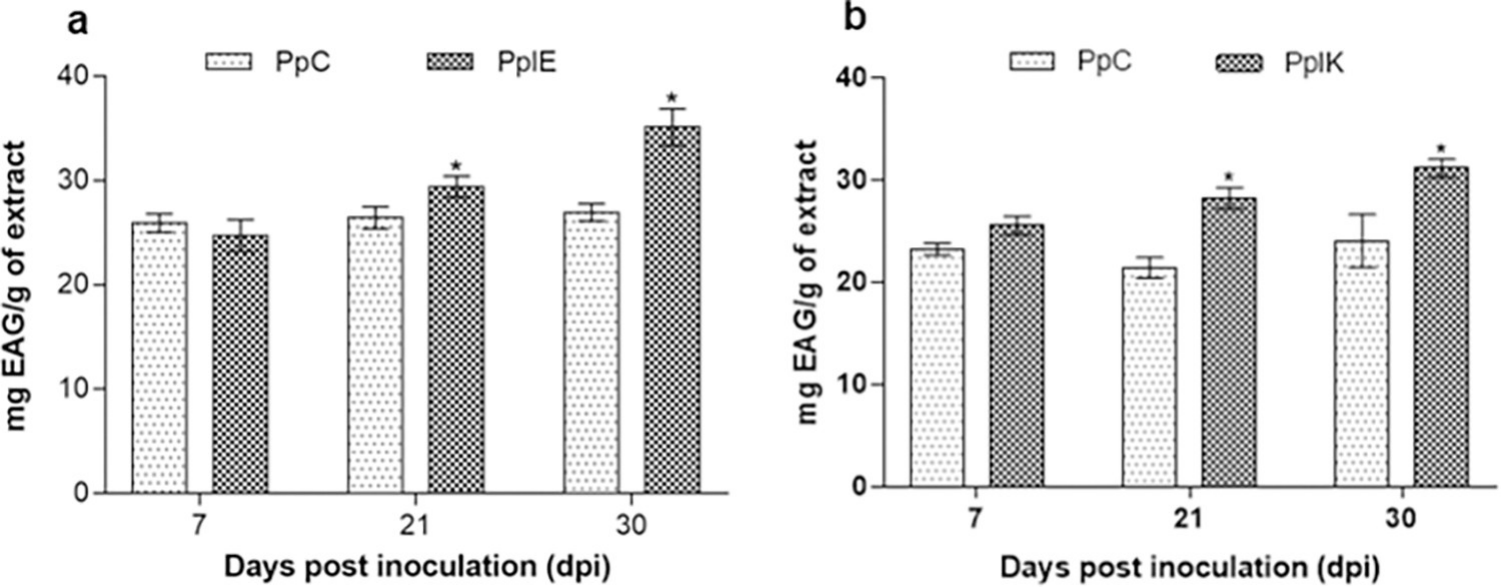
Total phenolic compounds of plants inoculated (n = 3) with *Enterobacter asburiae* (A) and *Klebsiella variicola* (B). *Statistically different according to Bonferroni test (*p* < 0.05). **dpi**: Days post inoculation. **PpC**: *P*. *pellucida* control. **PpIE**: *P*. *pellucida* inoculated with *Enterobacter asburiae*. **PpIK**: *P*. *pellucida* inoculated with *Klebsiella variicola*.

The concentration of total phenolic compounds in leaf extracts was improved by both inoculations. Plants treated with *E*. *asburiae* displayed an increase of 11.40% and 30.50% in comparison to control groups at 21 and 30 dpi (26.40–29.40 and 26.90–35.10 mg EAG/g of extract, respectively) (Fig 3A). *Peperomia pellucida* colonized by *K*. *variicola* had an increase in TPC of 31.20% at 21 dpi (21.50–28.20 mg) and of 30.0% at 30 dpi (24.0–31.20 mg EAG/g of extract) (Fig 3B).

### 3.5 Analysis of the volatile compounds

The analysis of the volatile compounds indicated 24 and 53 compounds for plants inoculated with *E*. *asburiae* and *K*. *variicola*, respectively (Tables 3 and 4). From 93.3 to 100.0% of the components were identified with a predominance of phenylpropanoids and derivatives (37.30–52.28%), followed by sesquiterpene hydrocarbons (39.28–49.42%). The components with contents above 2% were the phenylpropanoid dillapiole (16.72–24.39%), the phenylpropanoid derivative (ArC2) 2,4,5-trimethoxystyrene (18.03–31.35%), and the sesquiterpene hydrocarbons ishwarane (19.37–27.58%), β-caryophyllene (9.69–12.21%), β-elemene (0.83–3.54%) and (*E*,*E*)-α-farnesene (2.93–6.56%).

**Table 3:** Comparison of volatile compounds produced in leaves of *Peperomia pellucida* inoculated and non-inoculated with *Enterobacter asburiae*.

			7 dpi		21 dpi		30 dpi	
Compounds	RI(C)	RI(L)	PpIE	PpC	PpIE	PpC	PpIE	PpC
(*E*)-β-Ocimene	1042	1044^a^	0.05±0.07		0.45±0.39	0.27±0.19	0.71±0.38	0.24±0.34
4-Methyldecane	1053	1051^n^	0.24±0.10	0.08±0.11	0.13±0.10	0.15±0.05	0.30±0.03	0.15±0.04
*n*-Undecane	1103	1100^a^		0.15±0.21		0.16±0.18	0.13±0.11	
Hexyl butanoate	1194	1191^a^				0.08±0.12*		
*n*-Decanal	1208	1201^a^	0.65±0.20	0.60±0.10	1.02±0.45	0.53±0.11	1.26±0.21	0.81±0.38
Octyl acetate	1214	1211^a^	1.07±0.10	1.38±0.29	1.34±0.32	1.00±0.10	1.78±0.44	1.00±0.41
4,6-Dimethyldodecane	1281	1285^n^	0.14±0.05	0.15±0.02	0.05±0.04	0.05±0.05	0.14±0.02	0.08±0.03
Hexyl hexanoate	1386	1382^a^				0.06±0.08*		
Butanoic acid	1389	1381^n^				0.08±0.11*		
β-Elemene	1395	1389^a^	2.02±0.20	0.98±0.69	2.62±0.53	0.83±1.14	2.29±0.11	2.08±0.04
**β-Caryophyllene**	1424	1417^a^	10.31±0.23	11.43±1.11	10.14±0.77	11.44±1.11	10.37±0.91	11.70±0.88
α-Humulene	1459	1452^a^	0.31±0.05	0.28±0.04	0.51±0.20	0.53±0.20	0.41±0.02	0.42±0.03
**Ishwarane**	1469	1465^a^	24.85±0.72	27.51±0.20	26.06±1.39	27.44±1.21	23.11±0.20	27.58±0.36
Germacrene D	1487	1484^a^	0.82±0.09	0.67±0.34	0.94±0.20	1.49±0.57	0.79±0.06	1.07±0.05
Aristolochene	1490	1487^a^			0.08±0.11*			
Valencene	1499	1495^a^			0.40±0.56*			
γ-Amorphene	1499	1495^a^	0.59±0.12	0.26±0.19	0.47±0.38	0.46±0.37	0.70±0.03	0.70±0.01
Pentadecane	1503	1500^a^		0.05±0.04	0.06±0.05	0.07±0.06	0.10±0.00	0.06±0.04
(*E*,*E*)-α-Farnesene	1512	1505^a^	6.07±0.47	3.10±0.80	6.56±1.53	6.19±2.81	5.16±0.62	5.87±0.02
Myristicin	1525	1517^a^	1.29±0.22	0.25±0.20	0.43±0.43	0.31±0.44	1.09±0.56	0.90±0.13
**RI(C):** Retention index calculated; **RI(L):** Retention index of library; **a:** Adams; **n:** NIST. *Compounds with low representativity. **Identification tentative–See Section 4 for more details. **dpi**: Days post inoculation. **PpC**: *P*. *pellucida* control (non-inoculated). **PpIE**: *P*. *pellucida* inoculated with *Enterobacter asburiae* (n = 3).								

2,4,5-Trimethoxystyrene**	1566	1621^n^	28.44±1.67	29.58±1.27	27.57±2.10	25.21±2.13	31.35±0.98	26.09±0.84
Carotol	1604	1594^a^	0.36±0.03	0.35±0.15	0.52±0.34	0.73±0.16	0.68±0.06	0.87±0.03
**Dillapiole**	1629	1620^a^	22.01±1.23	22.45±0.14	19.87±1.11	18.96±0.98	19.57±1.19	20.31±0.27
Apiole	1685	1677^a^			0.12±0.12	0.10±0.07	0.06±0.05	0.05±0.07
Monoterpene hydrocarbons			0.05±0.00		0.45±0.00	0.27±0.00	0.71±0.00	0.24±0.00
Sesquiterpene hydrocarbons			44.97±8.25	44.23±9.40	47.78±8.03	48.38±9.19	42.83±7.68	49.42±9.17
Oxygenated sesquiterpenes			0.36±0.00	0.35±0.00	0.52±0.00	0.73±0.00	0.68±0.00	0.87±0.00
Phenylpropanoids and derivatives			51.74±11.58	52.28±12.49	47.99±12.03	44.58±11.16	52.07±13.13	47.35±11.55
Others			2.10±0.37	2.41±0.47	2.60±0.55	2.18±0.30	3.71±0.66	2.10±0.40
**Total (%)**			99.22	99.27	99.34	96.14	100.00	99.98
**RI(C):** Retention index calculated; **RI(L):** Retention index of library; **a:** Adams; **n:** NIST. *Compounds with low representativeness. **Identification tentative–See Section 4 for more details. **dpi**: Days post inoculation. **PpC**: *P*. *pellucida* control (non-inoculated). **PpIE**: *P*. *pellucida* inoculated with *Enterobacter asburiae* (n = 3).								

**Table 4:** Comparison of volatile compounds produced in leaves of *Peperomia pellucida* inoculated and non-inoculated with *Klebsiella variicola*.

			7 dpi		21 dpi		30 dpi	
Compounds	RI(C)	RI(L)	PpIK	PpC	PpIK	PpC	PpIK	PpC
*n*-Octane	814	800^a^	0.29±0.20	0.39±0.13	2.53±2.80	0.36±0.27	2.56±1.27	0.86±0.40
(2*E*)-Hexenal	841	846^a^	0.14±0.10	0.18±0.21		0.07±0.06		
2-Methyloctane	854	852^n^			0.29±0.30		0.23±0.19	0.10±0.06
*n*-Nonane	897	900^a^		0.07±0.10	1.12±1.58		1.45±0.65	0.51±0.42
Nonene	928	924^n^			0.20±0.29*			
Tetrahydrocitronellene	935	930 ^a^			0.06±0.09		0.39±0.15	0.08±0.06
4-Methylnonane	949	951^n^			0.05±0.07*			
Mesitylene	993	994^a^			0.10±0.14*		0.12±0.06*	
*n*-Decane	998	1000^a^			0.30±0.43		0.42±0.14	0.14±0.11
Acetophenone	1021	1029^n^						0.28±0.23*
(*E*)-β-Ocimene	1046	1044^a^	0.12±0.09	0.24±0.34	0.56±0.33	0.10±0.02	0.88±0.45	0.05±0.04
4-Methyldecane	1055	1051^n^	0.75±0.07	0.66±0.18	0.89±0.47	0.74±0.11	0.13±0.03	0.41±0.27
2-Methyldecane	1061	1051^n^	0.16±0.01	0.15±0.03	0.06±0.08	0.18±0.03		0.09±0.07
*n*-Octanol	1067	1063^a^		0.17±0.05	0.23±0.28		0.25±0.04	0.16±0.11
*n*-Undecane	1102	1100^a^	0.67±0.01	0.71±0.12	0.75±0.24	0.70±0.12	0.40±0.06	0.50±0.31
Naphthalene	1182	1178^a^	0.34±0.06	0.32±0.11	0.37±0.28	0.40±0.06		0.20±0.16
Hexyl butanoate	1191	1191^a^			0.07±0.05*		0.06±0.04*	
*n*-Decanal	1205	1201^a^	0.84±0.08	1.25±0.11	1.14±0.56	0.44±0.13	1.24±0.21	0.66±0.18
Caprylyl acetate	1210	1214^n^			0.82±1.16		0.70±0.99	0.56±0.46
Octyl acetate	1211	1211^a^	1.17±0.16	1.39±0.16	0.68±0.48	0.74±0.22	0.92±0.65	0.37±0.30
Isoamyl hexanoate	1252	1246^a^					0.11±0.10*	
**RI(C):** Retention index calculated; **RI(L):** Retention index of library; **a:** Adams; **n:** NIST. *Compounds with low representativeness. **Identification tentative–See Section 4 for more details. **dpi**: Days post inoculation. **PpC**: *P*. *pellucida* control (non-inoculated). **PpIK**: *P*. *pellucida* inoculated with *Klebsiella variicola* (n = 3).								

4,6-Dimethyldodecane	1280	1285^n^	0.44±0.07	0.39±0.13	0.44±0.33	0.48±0.07		0.24±0.24
Tridecane	1302	1300^a^			0.05±0.07*			
2,6,11-Trimethyldodecane	1326	1320^n^	0.16±0.03	0.13±0.05	0.16±0.12	0.17±0.03		0.09±0.07
Eugenol	1366	1357^f^		0.06±0.08	0.07±0.01			0.05±0.04
β-Bourbonene	1387	1387^a^	0.12±0.08	0.05±0.08		0.07±0.06		
Butanoic acid	1389	1381^a^	0.10±0.07	0.17±0.00	0.15±0.04		0.21±0.06	
1-Tetradecene	1392	1388^a^				0.05±0.04*		
β-Elemene	1394	1389^a^	2.68±0.36	2.88±0.32	3.06±0.07	2.98±0.11	3.49±0.44	3.54±0.46
Dodecanal	1408	1408^a^		0.06±0.00*	0.05±0.04*		0.13±0.06	0.09±0.07
Decyl acetate	1410	1407^a^	0.05±0.04	0.09±0.01	0.09±0.04		0.10±0.04	0.11±0.09
**β-Caryophyllene**	1422	1417^a^	12.14±0.15	12.21±0.83	9.69±0.55	11.34±0.55	11.05±0.52	10.86±0.40
β-Copaene	1432	1430^a^					0.10±0.07*	0.07±0.06*
Spirolepechinene	1446	1449^a^	0.08±0.06	0.06±0.08	0.11±0.02	0.07±0.05	0.25±0.06	0.17±0.08
α-Humulene	1456	1452^a^	0.84±0.14	0.96±0.13	0.84±0.02	0.89±0.04	1.15±0.16	1.09±0.17
**Ishwarane**	1466	1465^a^	23.77±1.24	23.65±0.88	19.37±2.77	26.14±0.46	20.79±1.98	21.73±3.60
β-Chamigrene	1471	1476^a^					0.11±0.08*	0.08±0.06*
Germacrene D	1484	1484^a^	1.13±0.24	1.18±0.27	1.20±0.14	1.24±0.09	1.56±0.36	1.52±0.22
Aristolochene	1487	1487^a^	0.40±0.15	0.46±0.10	0.41±0.03	0.48±0.04	0.64±0.05	0.57±0.07
γ-Amorphene	1495	1495^a^	0.40±0.56*					
Valencene	1496	1496^a^	1.12±0.79	1.63±0.14	1.51±0.22	1.76±0.01	1.86±0.10	1.82±0.05
Pentadecane	1499	1499^a^	0.30±0.04	0.31±0.03	0.25±0.03	0.40±0.06	0.32±0.09	0.35±0.04
(*E*,*E*)-α-Farnesene	1509	1505^a^	2.93±0.45	3.44±0.96	3.10±1.08	3.26±0.11	4.12±0.67	3.97±0.59
Myristicin	1522	1517^a^	1.35±0.37	1.58±0.33	1.30±0.21	1.08±0.30	1.00±0.22	1.14±0.04
**RI(C):** Retention index calculated; **RI(L):** Retention index of; **a:** Adams; **n:** NIST. *Compounds with low representativeness. **Identification tentative–See Section 4 for more details. **dpi**: Days post inoculation. **PpC**: *P*. *pellucida* control (non-inoculated). **PpIK**: *P*. *pellucida* inoculated with *Klebsiella variicola* (n = 3).								

β-Sesquiphellandrene	1525	1521^a^					0.06±0.04*	0.05±0.04*
2,4,5-Trimethoxystyrene**	1566	1621^n^	20.19±0.36	18.03±2.37	19.70±3.24	20.00±1.39	18.85±1.47	19.96±0.04
Hexanoic acid	1583	1580^n^					0.10±0.09*	0.07±0.06*
Caryophyllene oxide	1586	1582^a^	0.25±0.10	0.37±0.12	0.17±0.03	0.21±0.06	0.29±0.13	0.29±0.06
Carotol	1600	1594^a^	1.20±0.03	1.26±0.38	1.36±0.04	1.07±0.19	1.63±0.40	1.65±0.47
**Dillapiol**	1626	1620^a^	22.69±2.99	22.72±3.21	19.34±1.65	20.97±0.71	16.59±1.80	21.04±0.05
14-Hydroxy-9-*epi*-(*E*)-caryophyllene	1672	1668^a^		0.06±0.05	0.12±0.03		0.24±0.11	0.22±0.16
Apiole	1683	1677^a^	0.48±0.18	0.57±0.20	0.60±0.22	0.62±0.12	0.86±0.38	1.04±0.34
Monoterpene hydrocarbons			0.12±0.00	0.24±0.00	0.62±0.25	0.10±0.00	1.26±0.25	0.13±0.01
Sesquiterpene hydrocarbons			45.60±6.48	46.51±6.58	39.28±5.43	48.22±7.12	45.18±5.49	45.46±5.69
Oxygenated sesquiterpenes			1.44±0.48	1.69±0.51	1.64±0.57	1.28±0.43	2.16±0.64	2.16±0.66
Phenylpropanoids and derivatives			44.71±10.30	42.95±9.75	41.00±9.25	42.68±9.82	37.30±8.44	43.21±9.69
Others			5.39±0.32	6.44±0.39	10.80±0.56	4.74±0.21	9.46±0.63	5.77±0.22
**Total (%)**			97.26	97.84	93.35	97.02	95.36	96.68
**RI(C):** Retention index calculated; **RI(L):** Retention index of library; **a:** Adams; **n:** NIST. *Compounds with low representativeness. **Identification tentative–See Section 4 for more details. **dpi**: Days post inoculation. **PpC**: *P*. *pellucida* control (non-inoculated). **PpIK**: *P*. *pellucida* inoculated with *Klebsiella variicola* (n = 3).								

**Fig 4:**
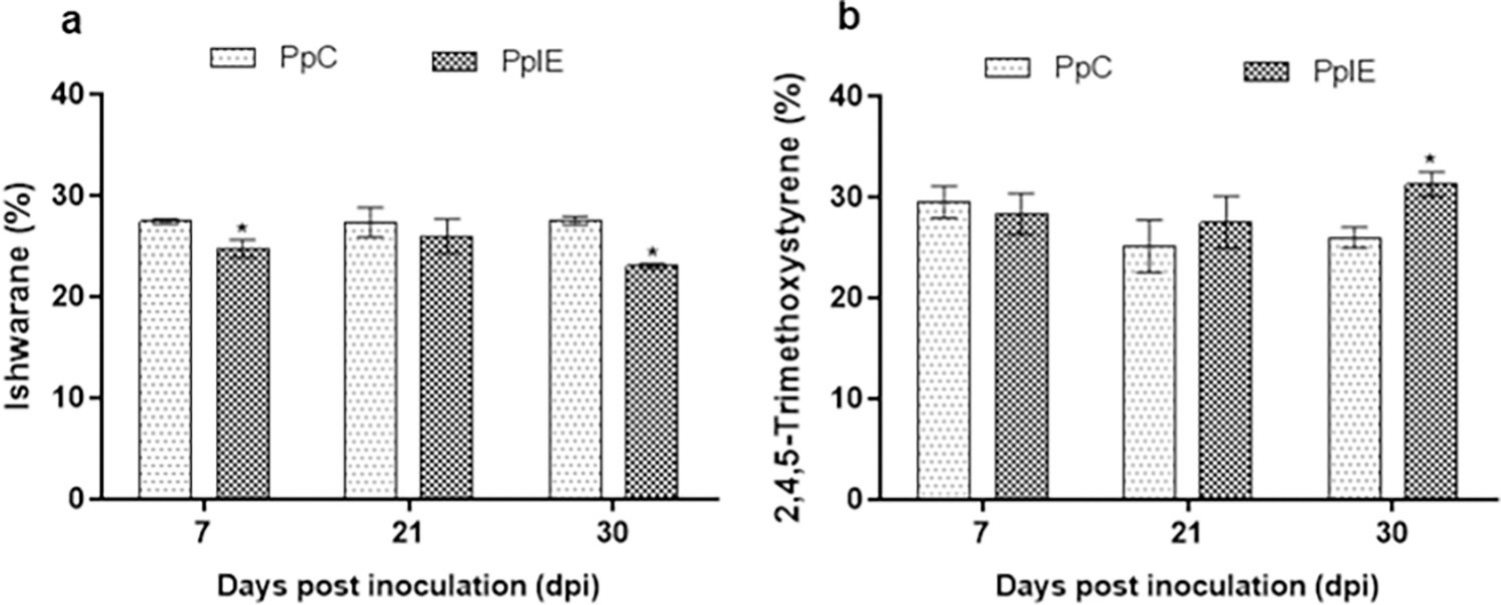
Variation in of percentage of ishwarane (A) and 2,4,5-trimethoxystyrene (B) in plants inoculated with *Enterobacter asburiae* according to Bonferroni test (p < 0.05), (n = 3). PpC: *P*. *pellucida* control. PpIE: *P*. *pellucida* inoculated with *Enterobacter asburiae*.

The percentage of the four major compounds dillapiole, 2,4,5-trimethoxystyrene, ishwarane and β-caryophyllene were submitted to an analysis of variance and a significant variation was observed only for plants inoculated with *E*. *asburiae*. This included the compound ishwarane which decreased at 7 (27.51–24.85%) and 30 dpi (27.58–23.11%), and 2,4,5-trimethoxystyrene, which was increased by 20.0% (26.09–31.35%) at 30 dpi (Fig 4A and 4B).

### 3.6 Multivariate analysis of the volatile composition of plants inoculated with *Enterobacter asburiae* and *Klebsiella variicola*

PCA (Principal Component Analysis) analyses were performed using as variables the total percentages of the majority classes and the constituents identified in the volatile fraction (content ≥ 2.0%).

**Fig 5:**
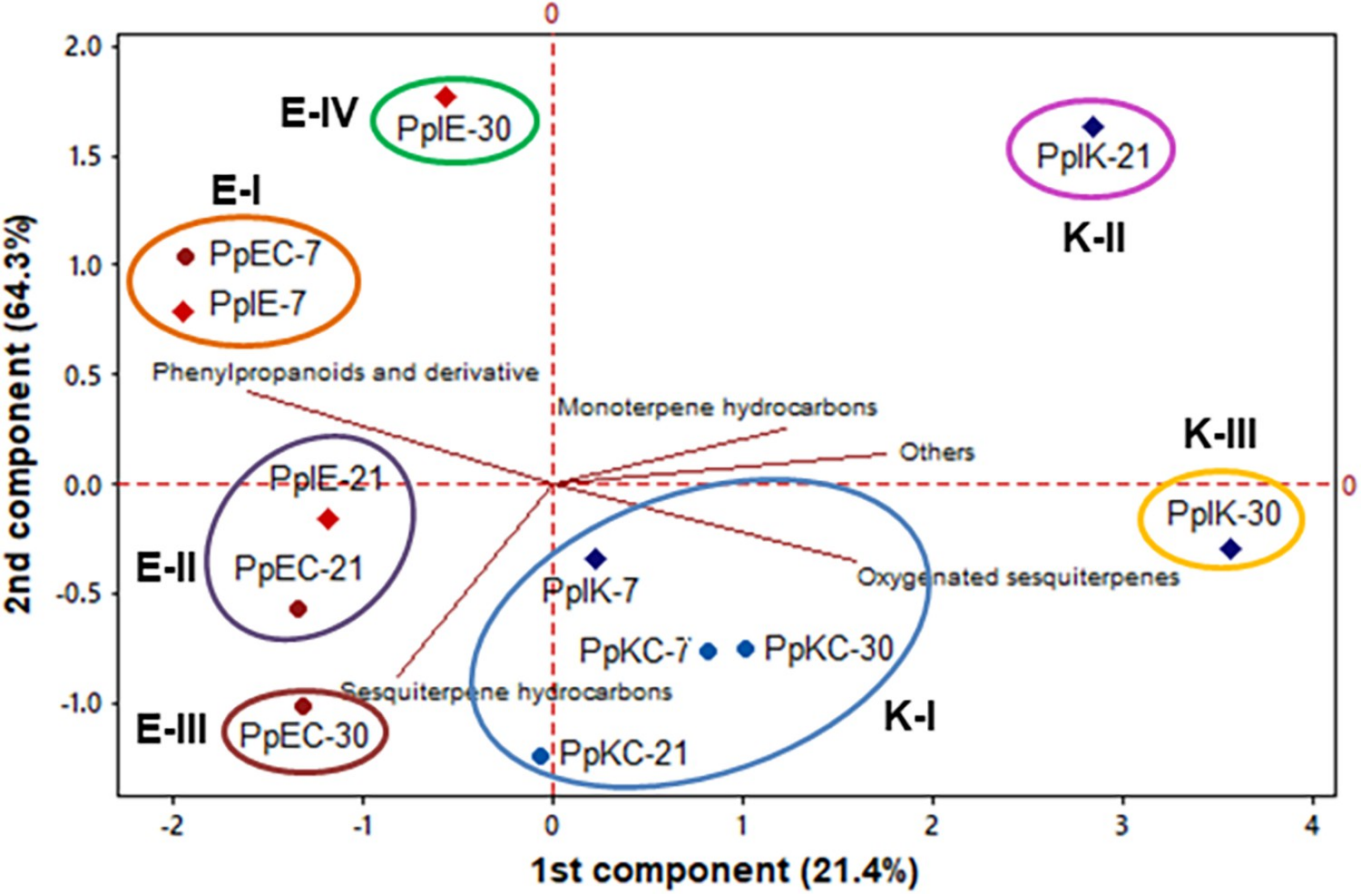
The bidimensional plot of the two components (PC1 and PC2) obtained in the PCA analysis of the classes of compound of controls and plants inoculated with *Enterobacter asburiae* and *Klebsiella variicola*.

PC1 and PC2 of the compound classes accounted for 85.7% of the data variance (Fig 5). PC1 separated samples inoculated with *E*. *asburiae* (negative loadings) from samples treated with *K*. *variicola* (positive loadings). Plants colonized by *E*. *asburiae* and the control samples were divided into four groups named from Groups E-I to IV and displayed similar loadings at 7 and 21 dpi. However, inoculated samples at 30 dpi (E-IV) had the greatest distance from its respective control group (E-III) with loadings of -0.57 in PC1 and 1.76 in PC2 which are mainly related to the reduction in the amount of sesquiterpenes hydrocarbons (49.42–42.83%) and increase in the phenylpropanoids and derivatives concentrations (47.35–52.07%) (Table 3).

Plants treated with *K*. *variicola* at 7 dpi and all control groups of this experiment formed a single group with negative loadings in PC2 and positive in PC1 (Group K-I). The samples PpIK-21 and PPIK-30 were individually separated from all the control samples and PPIK-7. The group PPIK-21 exhibited positive loadings in both PC1 and PC2 (2.84 and 1.63) while PPIK-30 had positive loading in PC1 and negative in PC2 (3.56 and -0.30), which are related to the increase in the concentration of monoterpene hydrocarbons (0.10–0.62% and 0.13–1.26%) and ‘other compounds’ (hydrocarbons, esters, ketones, etc.) (4.74–10.80% and 5.77–9.46%) in comparison to the controls, respectively. The sample PPIK-30 was also close to the Group K-I because of their similar content in oxygenated sesquiterpenes (Table 4).

**Fig 6:**
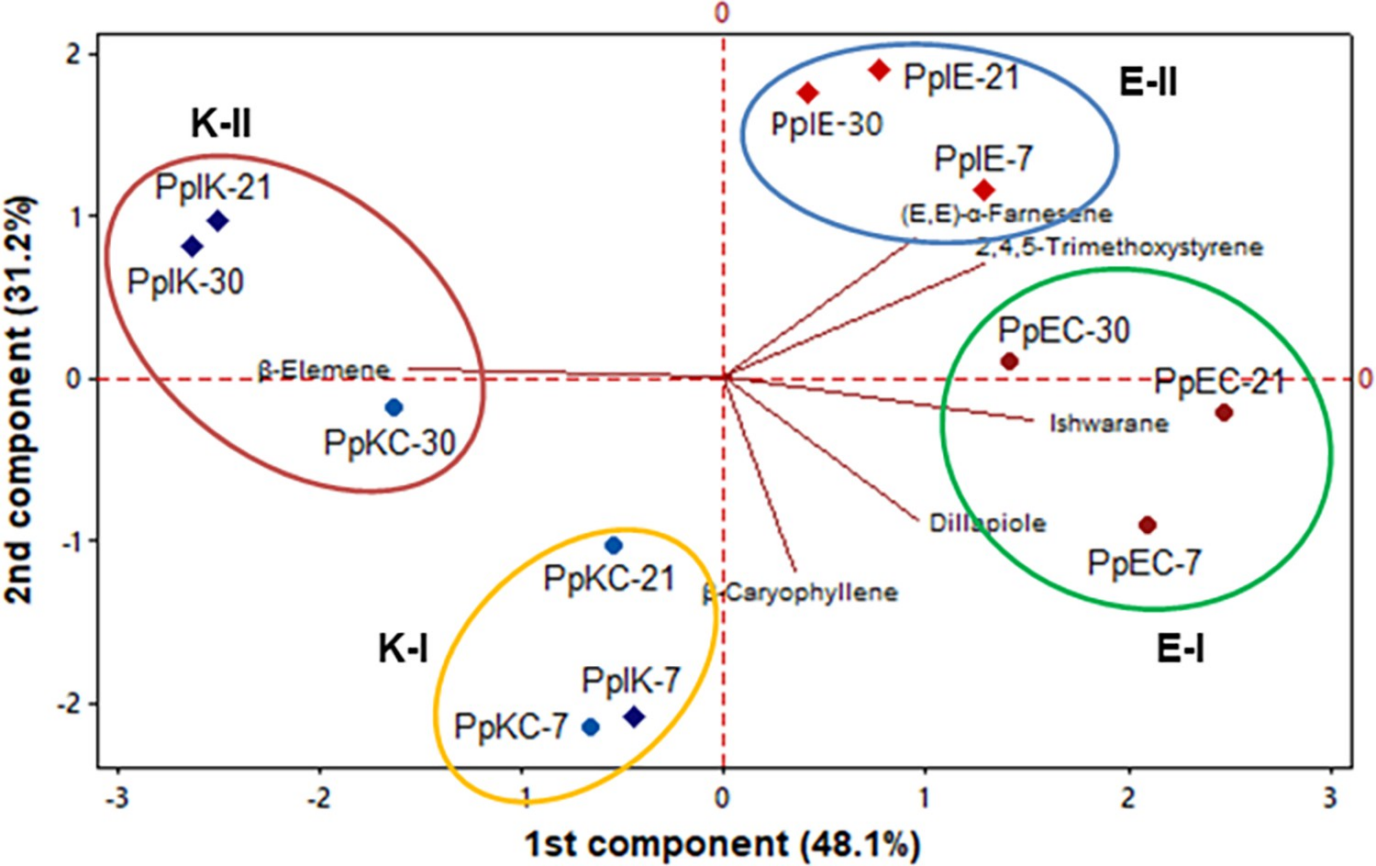
The bidimensional plot of the two components (PC1 and PC2) obtained in the PCA analysis of the compounds of controls and plants inoculated with *Enterobacter asburiae* and *Klebsiella variicola*.

PC1 and PC2 analysis of the volatile compounds (content ≥ 2.0%) comprised 79.3% of the total variability and separated plants colonized with *E*. *asburiae* and *K*. *variicola* in PC1 into positive and negative loadings, respectively (Fig 6).

The experiment carried out with *E*. *asburiae* effectively separated inoculated samples from the control samples. The Group E-I (PpC-7, PpC-21 and PpC-30) had a predominance of the phenylpropanoid dillapiole (22.45%, 22.33%, and 20.31%) and the sesquiterpene hydrocarbons ishwarane (27.5%, 27.4%, and 27.6%) and β-caryophyllene (11.43%, 11.44%, and 11.70%). Group E-II, composed by inoculated samples (PpIE-7, PpIE-21 and PpIE-30), was mainly characterized by an initial increase in the content of the sesquiterpenes hydrocarbon (*E*,*E*)-α-farnesene and decrease at 30 dpi (5.89–5.16%). In addition, the samples had an increase in the concentration of the phenylpropanoid derivative (ArC2) 2,4,5-trimethoxystyrene mainly at 30 dpi (26.09–31.35%), which corroborate with the results presented in the Section 3.5. These compounds had positive loadings in PC1 and PC2.

Plants colonized with *K*. *variicola* and its respective controls were distributed into two groups: Group K-I was composed of PpC-7, PpI-7 and PpC-21 and characterized by the predominance of β-caryophyllene (12.21%, 12.14%, and 11.34%) while Group K-II, consisting of PpI-21, PpC-30 and PpI-30, stood out mainly for the similar concentration of β-elemene (3.06%, 3.54%, and 3.49%). These groups’ formation indicates that there was no significant difference in the contents of the components of plants inoculated with *K*. *variicola*.

## 4. Discussion

The genera *Enterobacter* and *Klebsiella* are well-known for their potential for plant growth promotion and biocontrol of agricultural diseases [51–57]. *E*. *asburiae* was reported to improve maize [58], peppers, lettuces, cucumbers and tomatoes development [26] while *K*. *variicola* is well-known for promoting soybean [59], maize [60] and wheat growth [61]. Both *E*. *asburiae* and *K*. *variicola* indicated potential to increase sugarcane growth [25,28]. *E*. *asburiae* strain EM56 and *K*. *variicola* strain EM09 were both isolated from *S*. *amazonicum* rhizosphere and used to inoculate *P*. *pellucida* because PGPR can be applied in all groups of plants. However, these microorganisms may have different effects on plant development and secondary metabolism.

Bacteria inoculation caused significant variations in *P*. *pellucida* developmental parameters. Specimens colonized by *E*. *asburiae* had the number of leaves reduced at 30 dpi while leaf weight was increased at 21 and 30 dpi. *K*. *variicola* caused a rise in the number of leaves at 21 and 30 dpi and improved leaf weight at 30 dpi. Tomato seedlings (*Solanum lycopersicum* L.) treated with different *Streptomyces* spp. (Stm) strains had no changes in the number of leaves but displayed a rise in leaf weight at 30 dpi [62]. Likewise, the inoculation of *Bacillus subtilis* in *Ocimum basilicum* increased leaf fresh weight at 30 dpi [14].

Furthermore, *E*. *asburiae* inoculation increased the number of nodes at 21 and 30 dpi but had no significant effect on height. Plant symbiosis with *K*. *variicola* enhanced the number of nodes at 21 and 30 dpi and plant height in all days analyzed. Similarly, marjoram seedlings (*Origanum majorana* L.) treated with *Pseudomonas fluorescens* and *Bradyrhizobium* sp. exhibited an improvement of 33.80% and 23.20% in the number of nodes, respectively [23]. The association of *B*. *subtilis* with *O*. *basilicum* also improved plant height by 16 mm at 14 dpi [14]. Plants colonized by *E*. *asburiae* had root weight increased at 21 and 30 dpi, but root length was not affected. The second inoculation caused a growth in root length and weight at 21 dpi, but no significant changes were found at 30 dpi. *Vigna radiata* (L.) R.Wilczek colonized by *P*. *aeruginosa* and *B*. *subtilis* showed a growth of 84.6% and 61.9% on root length and 369.1% and 239.8% on its fresh weight at 35 dpi, respectively [63]. Hydroponic beans (*Vigna radiata)* associated with *Enterobacter sp*. P36 exhibited a raise of 64.20% in root weight [64].

These rhizobacteria potentials to improve plant growth can be explained by the presence of several genes with plant-beneficial functions. The genome of *K*. *variicola* was reported to contain *nif* cluster, indole-3-pyruvate decarboxylase (*ipdC)*, siderophore enterobactin synthesis genes (*ent*ABCDEF) and enterobactin exporter gene (*ent*S), and pyrroloquinoline quinone synthesis genes (*pqq*BCDEF), which are responsible for its N_2_ fixation, indole-3-acetic acid (IAA) production, siderophore production, and phosphate solubilization properties [27]. *E*. *asburiae* was also found to have genes involved in N_2_ fixation, auxin synthesis (*iaa* and *ipdC*), phosphorus metabolism and siderophore biosynthesis. All these genes together promote plant-bacteria communication and symbiosis [50,65].

The enzyme PAL plays an important role in inducing plant defense responses [66,67] since it is the first enzyme in the phenylpropanoid metabolic pathway which converts the amino acid phenylalanine into (*E*)-cinnamic acid [67,68]. Since it is involved in the regulation of this metabolic pathway, PAL can cause accumulation of lignins and phytoalexins that induce disease resistance [69]. The inoculation of *P*. *pellucida* with *E*. *asburiae* and *K*. *variicola* increased enzymatic activity mainly at 30 dpi (Fig 2). Similarly, *Mentha piperita* inoculated and co-inoculated with different rhizobacteria species showed a growth of approximately 300% in the enzyme activity [39].

Phenolic compounds are secondary metabolites produced by the shikimate pathway and pentose phosphate pathway through the metabolization of phenylpropanoids [70–72]. They are well known for their antioxidant, antibacterial, antifungal, and UV protection activities. They can also act as defense agents in plants [72,73]. Inoculated propagules showed a growth in TPC at 21 and 30 dpi (Fig 3). Likewise, specimens of chickpea (*Cicer arietinum* L.) inoculated and co-inoculated with *P*. *fluorescens* and *P*. *aeruginosa* displayed an increase in phenolic content in various growth stages [74]. These compounds have the potential to induce seed germination and improve plant development [75–79]. They can also contribute to plant defense against phytopathogens and can act as signaling molecules for symbiont recognition [39,80–82]. The increase in PAL activity and TPC may be related to both of their roles in inducing plant defense responses since colonization by symbiotic microorganisms may be initially recognized as a pathogen infection, causing a biotic stress [66,83].

The occurrence of dillapiole, 2,4,5-trimethoxystyrene, and β-caryophyllene as the major compounds of *P*. *pellucida* has been previously reported. For instance, EOs of plants collected in the northern region of Brazil had dillapiole (39.70–55.30%) and β-caryophyllene (10.70–14.3%) as some of their main components [1,7,9]. The compound 2,4,5-trimethoxystyrene was reported for *P*. *pellucida* collected in the Philippines [84], Belém [85] and São Paulo, in Brazil [86]. In this study, 2,4,5-trimethoxystyrene retention index (RI) of library (1621, NIST) was much higher than the RI calculated (1566), which probably happened because the library value was defined using a DB-1 capillary column. However, the RI values 1565 [87], 1551 [88] and 1552 [89] have also been reported for this compound. The indices found in these studies are much closer to our calculated RI, which explains this tentative compound identification (Tables 3 and 4).

The analysis of variance of the major compounds showed significant changes only for plants colonized by *E*. *asburiae* which had a decrease in the concentration of ishwarane and an increase in 2,4,5-trimethoxystyrene (Fig 4). The growth in the phenylpropanoid derivative content was an unexpected finding since monoterpene and sesquiterpene contents are usually the ones affected by microorganism inoculation [21,38,90]. The increase in this compound content may not have been reported for plant-microbe symbiosis, but 2,4,5-trimethoxystyrene could be involved in plant-defense since it has insecticidal activity [91].

The compound classes and the components with percentages ≥ 2.0% were submitted to multivariate analysis (Figs 5 and 6). Plants inoculated with *E*. *asburiae* were mainly characterized by the decrease and increase of sesquiterpene hydrocarbons and phenylpropanoids and derivatives at 30 dpi, respectively (Fig 5 and Table 3). The compound 2,4,5-trimethoxystyrene had positive loadings in both components (PC1 and PC2) and contributed the most in the separation of inoculated samples from control samples mainly at 30 dpi (Fig 6), which confirms the results expressed by the analysis of variance (Fig 4). Likewise, individuals colonized by *K*. *variicola* had a predominance of monoterpene hydrocarbons and ‘other compounds’ such as hydrocarbons, esters, ketones, and so on (Fig 5 and Table 4). The contents of compounds ≥ 2.0% were not affected by this bacterial colonization in comparison to the control groups (Fig 6).

The compound dillapiole is a phenylpropanoid that has antioxidant, antimicrobial, insecticidal, antitumor and anti-inflammatory activity [92–95]. Although it has been reported as the main component of *P*. *pellucida* EOs occurring in northern Brazil [1,7,9], plant colonization by *E*. *asburiae* and *K*. *variicola* did not affected its concentration. This probably happened because plant colonization by symbiotic microorganisms usually affects species rich in terpenes [14,21,38,90]. Similar effects have also been observed after herbivore attack [96–98]. These organisms affect the content of plant compounds by upregulating the expression of genes related to terpenoids, phenylpropanoids and other classes of compounds metabolic pathways [99].

Terpenes are characterized by having basic isoprene structures (C5) and are toxic substances that can stop herbivore attack [72]. Components of this group are usually related to plant defense mechanisms during colonization and infection [72,100] since they have insecticidal, fungicidal and antibacterial activity [101–104]. This study showed improvements in the concentrations of some classes of terpenes after bacteria symbiotic association with *P*. *pellucida*. It also indicated that plant colonization by rhizobacteria may increase phenylpropanoids contents since there was a rise in the concentration of the phenylpropanoid derivative 2,4,5-trimethoxystyrene.

## 5. Conclusions

The inoculation of *P*. *pellucida* with *E*. *asburiae* strain EM56 and *K*. *variicola* strain EM09 proved to be an efficient alternative to promote plant growth in this species since these microorganisms improved plant development. Furthermore, these bacteria increased PAL enzyme activity and total phenolic content which are both related to plant defense mechanisms during biotic stress.

*E*. *asburiae* inoculation caused an increase mainly in the content of 2,4,5-trimethoxystyrene, while *K*. *variicola* inoculation did not show any significant variations in the concentrations of the major compounds. Both inoculations affected the classes of terpenes, but only *E*. *asburiae* treatment increased the content of the class of phenylpropanoids and derivatives. These data show that the production of secondary metabolites in *P*. *pellucida* can be optimized by rhizobacteria inoculation, but factors such as the microorganism, the plant species and the plant chemical profile should be considered. The next step of this research will be the inoculation of both bacteria since it could improve even more plant growth and the production of secondary metabolites.

## Supporting information

S1 File (DOCX)

S2 File (XLSX)

S1 Graphical abstract (TIF)
