# Supplementary material for: Variation in Peperomia pellucida growth and secondary metabolism after rhizobacteria inoculation
Source: PLoS One. 2022 Jan 21;17(1):e0262794. doi: 10.1371/journal.pone.0262794 (PMC8785609; doi:10.1371/journal.pone.0262794)
Supplement: S1 File — (DOCX) [file pone.0262794.s001.docx]

**Supporting information S1. Supporting Tables.** This file contains supporting tables disclosing the minimal data set of plant growth promotion, PAL enzyme activity and total phenolic compounds.

10 Supporting Tables

**Table S1**. Developmental parameters of *Peperomia pellucida* after inoculation with *Enterobacter asburiae* at 7 dpi.

| **PpC - 7 dpi** | | | | | | **PpIE - 7 dpi** | | | | |
| --- | --- | --- | --- | --- | --- | --- | --- | --- | --- | --- |
| **Plant Number** | **1** | **2** | **3** | **Average** | **SD** | **1** | **2** | **3** | **Average** | **SD** |
| **Height (cm)** | 19.5 | 19.0 | 21.7 | 20.1 | 1.2 | 17.0 | 19.5 | 18.5 | 18.3 | 1.0 |
| **Nodes (n^o^)** | 16.0 | 16.0 | 19.0 | 17.0 | 1.2 | 20.0 | 18.0 | 21.0 | 19.7 | 1.2 |
| **Leaves (n^o^)** | 27.0 | 24.0 | 22.0 | 24.3 | 1.8 | 30.0 | 20.0 | 29.0 | 26.3 | 4.5 |
| **Root (cm)** | 10.5 | 10.5 | 8.5 | 9.8 | 0.8 | 11.0 | 11.0 | 12.6 | 11.5 | 0.8 |
| **Fresh weight –root (g)** | 0.1 | 0.1 | 0.3 | 0.2 | 0.1 | 0.2 | 0.2 | 0.4 | 0.3 | 0.1 |
| **Fresh weight – leaves (g)** | 0.6 | 0.6 | 0.7 | 0.6 | 0.1 | 1.3 | 1.1 | 1.9 | 1.4 | 0.3 |

**Legend:** **dpi**: days post inoculation. **PpC**: *P. pellucida* control*.* **PpIE**: *P. pellucida* inoculated with *Enterobacter asburiae.*

**Table S2.** Developmental parameters of *Peperomia pellucida* development after inoculation with *Enterobacter asburiae* at 21 dpi.

| **PpC - 21 dpi** | | | | | | **PpIE - 21 dpi** | | | | |
| --- | --- | --- | --- | --- | --- | --- | --- | --- | --- | --- |
| **Plant Number** | **1** | **2** | **3** | **Average** | **SD** | **1** | **2** | **3** | **Average** | **SD** |
| **Height (cm)** | 38.0 | 36.0 | 36.5 | 36.8 | 0.8 | 39.3 | 33.0 | 31.50 | 34.6 | 3.4 |
| **Nodes (n^o^)** | 49.0 | 44.0 | 45.0 | 46.0 | 2.2 | 67.5 | 69.0 | 66.00 | 67.5 | 1.2 |
| **Leaves (n^o^)** | 66.0 | 76.0 | 73.0 | 71.7 | 4.2 | 89.5 | 89.0 | 90.00 | 89.5 | 0.4 |
| **Root (cm)** | 14.0 | 11.5 | 14.5 | 13.3 | 1.3 | 13.5 | 16.0 | 15.00 | 14.8 | 1.0 |
| **Fresh weight –root (g)** | 0.9 | 0.8 | 0.9 | 0.9 | 0.1 | 0.9 | 1.9 | 1.71 | 1.5 | 0.4 |
| **Fresh weight – leaves (g)** | 3.0 | 2.8 | 3.0 | 2.9 | 0.1 | 3.7 | 3.9 | 4.0 | 3.9 | 0.1 |

**Legend:** **dpi**: days post inoculation. **PpC**: *P. pellucida* control*.* **PpIE**: *P. pellucida* inoculated with *Enterobacter asburiae.*

**Table S3.** Developmental parameters of *Peperomia pellucida* development after inoculation with *Enterobacter asburiae* at 30 dpi.

| **PpC - 30 dpi** | | | | | | **PpIE - 30 dpi** | | | | |
| --- | --- | --- | --- | --- | --- | --- | --- | --- | --- | --- |
| **Plant Number** | **1** | **2** | **3** | **Average** | **DP** | **1** | **2** | **3** | **Average** | **DP** |
| **Height (cm)** | 34.0 | 40.0 | 36.5 | 36.8 | 2.5 | 36.5 | 37.5 | 36.0 | 36.7 | 0.6 |
| **Nodes (n^o^)** | 43.0 | 36.0 | 39.5 | 39.5 | 2.9 | 54.0 | 54.0 | 49.0 | 52.3 | 2.4 |
| **Leaves (n^o^)** | 71.0 | 79.0 | 75.0 | 75.0 | 3.3 | 64.0 | 69.0 | 64.0 | 65.7 | 2.4 |
| **Root (cm)** | 16.5 | 13.0 | 15.0 | 14.8 | 1.4 | 18.0 | 14.0 | 13.5 | 15.2 | 2.0 |
| **Fresh weight –root (g)** | 0.5 | 0.8 | 0.70 | 0.7 | 0.1 | 1.1 | 1.5 | 1.3 | 1.3 | 0.2 |
| **Fresh weight – leaves (g)** | 2.0 | 1.4 | 2.1 | 1.8 | 0.3 | 2.6 | 3.0 | 3.1 | 2.9 | 0.2 |

**Legend:** **dpi**: days post inoculation. **PpC**: *P. pellucida* control*.* **PpIE**: *P. pellucida* inoculated with *Enterobacter asburiae.*

**Table S4.** Developmental parameters of *Peperomia pellucida* development after inoculation with *Klebsiella variicola* at 7 dpi.

| **PpC - 7 dpi** | | | | | | **PpIK - 7 dpi** | | | | |
| --- | --- | --- | --- | --- | --- | --- | --- | --- | --- | --- |
| **Plant Number** | **1** | **2** | **3** | **Average** | **SD** | **1** | **2** | **3** | **Average** | **SD** |
| **Height (cm)** | 16 | 18 | 18.5 | 17.5 | 1.1 | 19.5 | 20 | 22.5 | 20.7 | 1.3 |
| **Nodes (n^o^)** | 15 | 18 | 18 | 17.0 | 1.4 | 24 | 23 | 21 | 22.7 | 1.2 |
| **Leaves (n^o^)** | 26 | 24 | 22 | 24.0 | 1.6 | 32 | 31 | 32 | 31.7 | 0.5 |
| **Root (cm)** | 8.5 | 8.5 | 8 | 8.3 | 0.2 | 10.5 | 10.5 | 10.3 | 10.4 | 0.1 |
| **Fresh weight –root (g)** | 0.19 | 0.20 | 0.14 | 0.2 | 0.0 | 0.40 | 0.42 | 0.26 | 0.4 | 0.1 |
| **Fresh weight – leaves (g)** | 1.24 | 1.06 | 0.99 | 1.1 | 0.1 | 2.10 | 1.73 | 1.78 | 1.9 | 0.2 |

**Legend:** **dpi**: days post inoculation. **PpC**: *P. pellucida* control*.* **PpIK**: *P. pellucida* inoculated with *Klebsiella variicola..*

**Table S5.** Developmental parameters of *Peperomia pellucida* development after inoculation with *Klebsiella variicola* at 21 dpi.

| **PpC - 21 dpi** | | | | | | **PpIK - 21 dpi** | | | | |
| --- | --- | --- | --- | --- | --- | --- | --- | --- | --- | --- |
| **Plant Number** | **1** | **2** | **3** | **Average** | **SD** | **1** | **2** | **3** | **Average** | **SD** |
| **Height (cm)** | 19.5 | 20.0 | 19.8 | 19.8 | 0.2 | 25.5 | 26.5 | 25.5 | 25.8 | 0.5 |
| **Nodes (n^o^)** | 47.0 | 32.0 | 50.0 | 43.0 | 7.9 | 54.0 | 69.0 | 74.0 | 65.7 | 8.5 |
| **Leaves (n^o^)** | 50.0 | 57.0 | 54.0 | 53.7 | 2.9 | 68.0 | 67.0 | 79.0 | 71.3 | 5.4 |
| **Root (cm)** | 11.0 | 7.5.0 | 12.0 | 10.2 | 1.9 | 14.0 | 15.4 | 11.5 | 13.6 | 1.6 |
| **Fresh weight –root (g)** | 0.2 | 0.2 | 0.2 | 0.2 | 0.0 | 0.7 | 0.5 | 0.6 | 0.6 | 0.1 |
| **Fresh weight – leaves (g)** | 2 | 1.8 | 2.1 | 2.0 | 0.1 | 3.1 | 2.08 | 3.3 | 2.8 | 0.5 |

**Legend:** **dpi**: days post inoculation. **PpC**: *P. pellucida* control*.* **PpIK**: *P. pellucida* inoculated with *Klebsiella variicola..*

**Table S6.** Developmental parameters of *Peperomia pellucida* development after inoculation with *Klebsiella variicola* at 30 dpi.

| **PpC - 30 dpi** | | | | | | **PpIK - 30 dpi** | | | | |
| --- | --- | --- | --- | --- | --- | --- | --- | --- | --- | --- |
| **Plant Number** | **1** | **2** | **3** | **Average** | **SD** | **1** | **2** | **3** | **Average** | **SD** |
| **Height (cm)** | 26.0 | 25.7 | 28.7 | 26.8 | 1.3 | 33.0 | 34.0 | 33.5 | 33.5 | 0.4 |
| **Nodes (n^o^)** | 50.0 | 35.0 | 34.0 | 39.7 | 7.3 | 72.0 | 64.0 | 73.0 | 69.7 | 4.0 |
| **Leaves (n^o^)** | 77.0 | 63.0 | 89.0 | 76.3 | 10.6 | 95.0 | 88.3 | 91.0 | 91.4 | 2.7 |
| **Root (cm)** | 11.0 | 10.8 | 11.9 | 11.2 | 0.5 | 13.0 | 14.0 | 13.0 | 13.3 | 0.5 |
| **Fresh weight –root (g)** | 0.5 | 0.7 | 0.5 | 0.6 | 0.1 | 1.1 | 0.6 | 0.7 | 0.8 | 0.2 |
| **Fresh weight – leaves (g)** | 2.8 | 2.7 | 2.7 | 2.8 | 0.1 | 5.3 | 3.5 | 3.9 | 4.2 | 0.8 |

**Legend:** **dpi**: days post inoculation. **PpC**: *P. pellucida* control*.* **PpIK**: *P. pellucida* inoculated with *Klebsiella variicola..*

**Table S7.** PAL enzyme activity of plants inoculated (n=3) with *Enterobacter asburiae.*

|  | **PpC** | | | | | **PpIE** | | | | |
| --- | --- | --- | --- | --- | --- | --- | --- | --- | --- | --- |
| **dpi** | **1** | **2** | **2** | **Average** | **SD** | **1** | **2** | **3** | **Average** | **SD** |
| **07** | 24.0 | 23.5 | 22.3 | 23.3 | 0.7 | 24.0 | 24.2 | 26.6 | 24.9 | 1.2 |
| **21** | 25.8 | 22.7 | 22.8 | 23.9 | 1.3 | 31.6 | 29.1 | 31.8 | 30.8 | 1.2 |
| **30** | 27.6 | 27.1 | 32.2 | 29.0 | 2.3 | 42.2 | 39.6 | 38.3 | 40.0 | 1.6 |

**Legend:** **dpi**: days post inoculation. **PpC**: *P. pellucida* control*.* **PpIE**: *P. pellucida* inoculated with *Enterobacter asburiae.* These values are given in µU/mL.

**Table S8.** PAL enzyme activity of plants inoculated (n=3) with *Klebsiella variicola.*

|  | **PpC** | | | | | **PpIK** | | | | |
| --- | --- | --- | --- | --- | --- | --- | --- | --- | --- | --- |
| **dpi** | **1** | **2** | **2** | **Average** | **SD** | **1** | **2** | **3** | **Average** | **SD** |
| **07** | 19.0 | 20.2 | 15.8 | 18.4 | 1.9 | 27.1 | 23.3 | 24.9 | 25.1 | 1.6 |
| **21** | 17.8 | 19.9 | 15.4 | 17.7 | 1.9 | 21.1 | 21.0 | 24.1 | 22.0 | 1.4 |
| **30** | 16.7 | 19.3 | 21.8 | 19.3 | 2.1 | 29.7 | 31.6 | 28.4 | 29.9 | 1.3 |

**Legend:** **dpi**: days post inoculation. **PpC**: *P. pellucida* control*.* **PpIK**: *P. pellucida* inoculated with *Klebsiella variicola.* These values are given in µU/mL.

**Table S9.** Total phenolic compounds of plants inoculated (n=3) with *Enterobacter asburiae.*

|  | **PpC** | | | | | **PpIE** | | | | |
| --- | --- | --- | --- | --- | --- | --- | --- | --- | --- | --- |
| **dpi** | **1** | **2** | **2** | **Average** | **SD** | **1** | **2** | **3** | **Average** | **SD** |
| **07** | 26.3 | 24.9 | 26.5 | 25.9 | 0.7 | 24.0 | 26.5 | 23.7 | 24.7 | 1.3 |
| **21** | 27.6 | 25.7 | 26.0 | 26.4 | 0.9 | 28.2 | 29.9 | 30.1 | 29.4 | 0.8 |
| **30** | 26.8 | 27.9 | 26.2 | 26.9 | 0.7 | 33.1 | 36.2 | 36.0 | 35.1 | 1.4 |

**Legend:** **dpi**: days post inoculation. **PpC**: *P. pellucida* control*.* **PpIE**: *P. pellucida* inoculated with *Enterobacter asburiae.* These values are given in mg EAG/g of extract.

**Table S10.** Total phenolic compounds of plants inoculated (n=3) with *Klebsiella variicola.*

|  | **PpC** | | | | | **PpIK** | | | | |
| --- | --- | --- | --- | --- | --- | --- | --- | --- | --- | --- |
| **dpi** | **1** | **2** | **2** | **Average** | **SD** | **1** | **2** | **3** | **Average** | **SD** |
| **07** | 23.2 | 23.8 | 22.7 | 23.2 | 0.5 | 25.9 | 26.3 | 24.5 | 25.6 | 0.8 |
| **21** | 20.6 | 22.6 | 21.2 | 21.4 | 0.8 | 28.9 | 28.7 | 27.0 | 28.2 | 0.9 |
| **30** | 21.0 | 25.6 | 25.5 | 24.1 | 2.1 | 31.3 | 30.3 | 32.0 | 31.2 | 0.7 |

**Legend:** **dpi**: days post inoculation. **PpC**: *P. pellucida* control*.* **PpIK**: *P. pellucida* inoculated with *Klebsiella variicola..* These values are given in mg EAG/g of extract.
