# Supplementary figures and images for: Variation in Peperomia pellucida growth and secondary metabolism after rhizobacteria inoculation
Source: PLoS One. 2022 Jan 21;17(1):e0262794. doi: 10.1371/journal.pone.0262794 (PMC8785609; doi:10.1371/journal.pone.0262794)

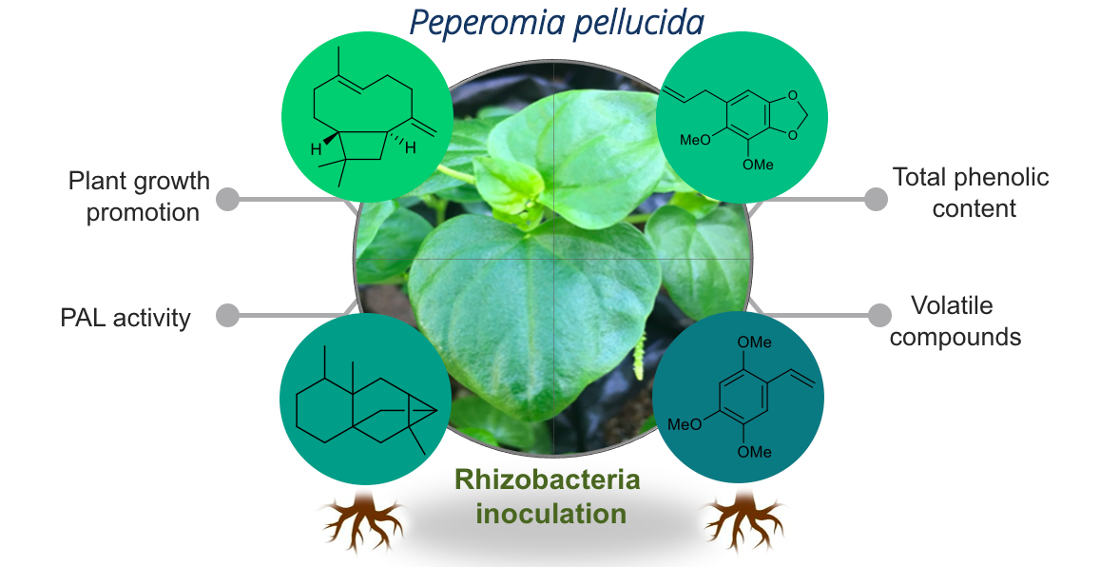

Supplement: S1 Graphical abstract — (TIF) [file pone.0262794.s003.tif]
